# Genetic Analysis of Grain Filling Rate Using Conditional QTL Mapping in Maize

**DOI:** 10.1371/journal.pone.0056344

**Published:** 2013-02-18

**Authors:** Zhanhui Zhang, Zonghua Liu, Zitian Cui, Yanmin Hu, Bin Wang, Jihua Tang

**Affiliations:** College of Agronomy/Key Laboratory of Physiological Ecology and Genetic Improvement of Food Crops in Henan Province, Henan Agricultural University, Zhengzhou, China; University of Nottingham, United Kingdom

## Abstract

The grain filling rate (GFR) is an important dynamic trait that determines the final grain yield and is controlled by a network of genes and environment factors. To determine the genetic basis of the GFR, a conditional quantitative trait locus (QTL) analysis method was conducted using time-related phenotypic values of the GFR collected from a set of 243 immortalized F_2_ (IF_2_) population, which were evaluated at two locations over 2 years. The GFR gradually rose in the 0–15 days after pollination (DAP) and 16–22 DAP, reaching a maximum at 23–29 DAP, and then gradually decreasing. The variation of kernel weight (KW) was mainly decided by the GFR, and not by the grain filling duration (GFD). Thirty-three different unconditional QTLs were identified for the GFR at the six sampling stages over 2 years. Among them, QTLs *qGFR7b*, *qGFR9* and *qGFR6d* were identified at the same stages at two locations over 2 years. In addition, 14 conditional QTLs for GFR were detected at five stages. The conditional QTL *qGFR7c* was identified at stage V|IV (37–43 DAP) at two locations over 2 years, and *qGFR7b* was detected at the sixth stage (44–50 DAP) in all four environments, except at Anyang location in 2009. QTLs *qQTL7b* and *qQTL6f* were identified by unconditional and conditional QTL mapping at the same stages, and might represent major QTLs for regulating the GFR in maize in the IF_2_ population. Moreover, most of the QTLs identified were co-located with QTLs from previous studies that were associated with GFR, enzyme activities of starch synthesis, soluble carbohydrates, and grain filling related genes. These results indicated that the GFR is regulated by many genes, which are specifically expressed at different grain filling stages, and the specific expression of the genes between 16–35 DAP might be very important for deciding the final kernel weight.

## Introduction

Grain yield has been a main target in cereal breeding, especially for maize (*Zea mays* L.), a critical source of food, fuel, feed, and fiber worldwide. [1] In maize, grain yield can be defined as the product of kernel sink capacity and grain filling efficiency, [2] and the GFR is regulated by multi-genes or by QTLs, as well as cultivation conditions, showing complex dynamic changes. To dissect the genetic bases of kernel development, certain genes corresponding to grain size or kernel development in maize, such as *rgf1*, *sh1*, *sh2*, *dek1*, *mn1*, and *CNR1*, have been cloned. [3]–[9] However, because of the difficulty in measuring natural variations in GFR, the molecular roles of genes or QTLs specifically expressed during grain filling have not been fully elucidated.

In cereal crops, grain filling is a critical and dynamic process that determines final grain yield. It depends on carbohydrates derived from two different sources: from photosynthesis in the leaf during the grain filling procedure and from accumulated nonstructural carbohydrates in culms and leaf sheaths. [10] The final kernel weight is mainly determined by the grain filling procedure. [11] In the field, the duration of grain filling is affected by changes in plant density and temperature, whereas the GFR is relative steady. [12] For maize, the final grain weight achieved by maize kernels is largely genetically determined. [13] However, factors such as assimilate availability, [14] the ‘sink capacity’ of an individual kernel, [12] kernel water content, [15], [16] leaf nitrogen dynamics, [10] related enzyme activity, [4] drought, [17] or high temperature [18] affect the GFR or GFD, limiting the achievement of maximum kernel weight.

For convenience, the grain filling procedure has been partitioned into three phases: the lag phase, the effective grain filling period and the maturation drying phase. [15] The lag phase is a period of active cell division, followed by differentiation and DNA endoreduplication, with almost no dry matter accumulation. During this phase, the GFR is low. [19] At the end of the lag phase, the GFR starts to rise and reaching its maximum value in the middle of the effective grain filling period. [15] In the effective grain filling period, the GFR and the duration of the effective grain filling period determine the final weight. [20] After reaching the maximum, the GFR gradually decreases, and the final kernel weight is achieved during the maturation drying phase. [15], [21] Although there are many factors that could affect the GFR during the three phases, the genotype has the most important role in affecting the GFR in cereal crops. [22], [23] Under these circumstances, the GFR shows a logistic curve during the grain filling procedure.

Recently, conditional QTL mapping has been used to dissect the genetic architecture of important quantitative traits in maize, such as plant height [24] and enzyme activity during grain development. [4] Although the GFR is an important developmental trait that directly decides the final grain yield, only three genes related to grain filling in maize and rice have been cloned: *rgf1*, *GS5* and *GIF1*. [3], [11], [25] The GFR is an important factor that decides grain yield in maize; however, its genetic basis is unclear. In this study, a set of immortalized F_2_ (IF_2_) maize plants was used to dissect the genetic basis of the GFR using conditional and unconditional QTL mapping. The goal of this study was to: (1) dissect the genetic basis of the GFR in maize, and (2) identify the unconditional and conditional QTLs that controlled the GFR in the different processes of carbohydrates synthesis.

## Results

### Climate Conditions in the Two Locations

**Figure 1:**
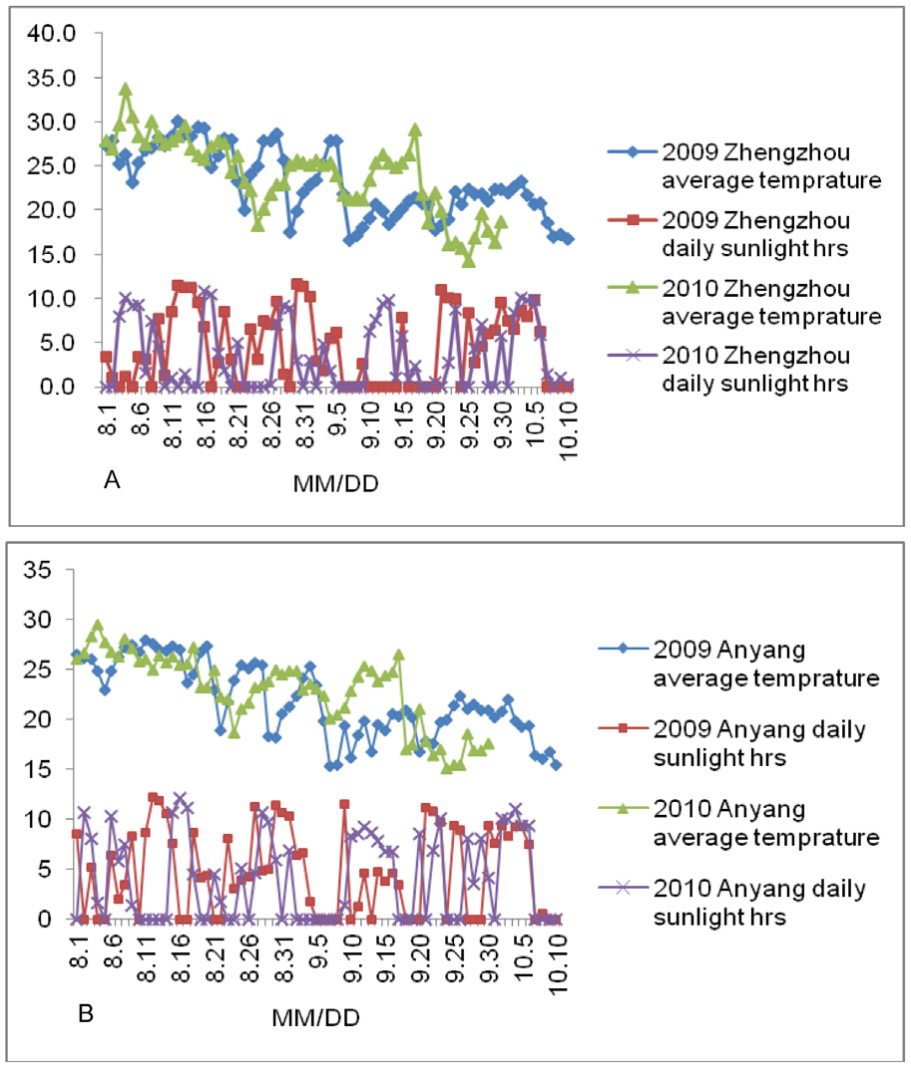
Two main climate factors in maize grain filling duration in 2009–2010 at Zhengzhou (a) and Anyang (b).

Temperature and sunlight conditions during the maize grain filling duration of the IF_2_ population across 2 years are shown in Fig. 1a and b. The results of variance analysis showed that the average temperature and sunlight were significant different between the 2 locations (at P = 0.01 significance level), there were large differences at according stages during grain filling duration in related climate factors between the two years at any one location. During grain filling duration at Zhengzhou location, the average of temperature in the IF_2_ population were 23.1°C and 24.0°C, and the average of daily sunlight were 4.2 hrs and 3.8 hrs in 2009 and 2010, respectively. In Anyang, the average temperature in the IF_2_ population was 21.9°C and 22.9°C, and the average of daily sunlight was 4.6 hrs and 4.1 hrs in 2009 and 2010, respectively.

### Variations in GFR

For the two parents (Table 1), the average GFR increased over the initial two or three sampling stages (0–22 DAP in 2009 and 0–29 DAP in 2010), and then decreased over the next one or two sampling stages (30–36 DAP in 2010, 23–36 DAP at Zhengzhou and 23–29 DAP at Anyang in 2009), as did that of the hybrid Nongda 108. Comparing the hybrid and its parents, the maximum GFR of the hybrid in almost all environments was higher than that of the two parents, and the KW of the hybrid was also higher than that of both parents.

**Table 1:** Performance of grain filling rate, grain filling duration and final kernel weight in the immortalized F_2_ population at two locations.

Year	Location	Population		KW^a^	GFD^b^	GFR^c^ mg °Cd^−1^ kernel^−1^					
				g 100^−1^ kernel^−1^	°Cd	I	II	III	IV	V	VI
2009	Zhengzhou	P_1_	Mean	28.30	1169.7	0.11	0.38	0.36	0.14	0.29	0.28
		P_2_	Mean	24.98	1163.4	0.08	0.42	0.367	0.152	0.377	0.251
		F_1_	Mean	29.52	1192.7	0.10	0.43	0.29	0.19	0.44	0.20
		IF_2_	Mean±SD	23.87±0.19	1178.2±0.8	0.06±0.001	0.34±0.01	0.36±0.01	0.28±0.01	0.26±0.01	0.21±0.01
			Range	14.93–35.55	1146.8–1207.2	0.03–0.13	0.11–0.64	0.12–0.64	0.01–0.56	0.01–0.63	0.02–0.65
	Anyang	P_1_	Mean	27.99	1006.1	0.10	0.51	0.24	0.48	0.34	0.23
		P_2_	Mean	23.82	1026.8	0.10	0.47	0.32	0.36	0.24	0.14
		F_1_	Mean	29.15	1036.4	0.11	0.53	0.19	0.44	0.42	0.24
		IF_2_	Mean±SD	23.88±0.19	1032.8±0.8	0.07±0.001	0.32±0.01	0.39±0.01	0.31±0.01	0.28±0.01	0.22±0.01
			Range	16.32–34.36	937.7–1051.9	0.04–0.15	0.09–0.67	0.12–0.70	0.04–0.71	0.01–0.70	0.01–0.66
2010	Zhengzhou	P_1_	Mean	36.38	1057.3	0.07	0.26	0.59	0.24	0.21	0.20
		P_2_	Mean	24.71	1097.4	0.08	0.36	0.43	0.07	0.39	0.22
		F_1_	Mean	28.32	1176.2	0.08	0.38	0.40	0.33	0.35	0.13
		IF_2_	Mean±SD	24.46±0.22	1136.4±3.6	0.06±0.001	0.31±0.01	0.34±0.01	0.29±0.01	0.27±0.01	0.25±0.01
			Range	17.64–35.54	938.0–1200.9	0.03–0.13	0.17–0.58	0.16–0.57	0.06–0.64	0.06–0.58	0.04–0.76
	Anyang	P_1_	Mean	36.67	987.7	0.08	0.35	0.48	0.21	0.41	0.14
		P_2_	Mean	25.13	1060.2	0.08	0.35	0.41	0.33	0.38	0.06
		F_1_	Mean	28.93	1096.5	0.09	0.25	0.52	0.34	0.42	0.26
		IF_2_	Mean±SD	24.38±0.20	1032.1±3.2	0.06±0.001	0.31±0.01	0.35±0.01	0.33±0.01	0.31±0.01	0.26±0.01
			Range	17.29–34.41	743.6–1108.3	0.03–0.12	0.15–0.66	0.18–0.71	0.04–0.65	0.01–0.67	0.01–0.74

Note: ^a^The kernel weight in the IF_2_ population;

b The total thermal time from pollination to the last sampling on average in the IF_2_ population;

c The average grain filling rate in the IF_2_ population.

**Figure 2:**
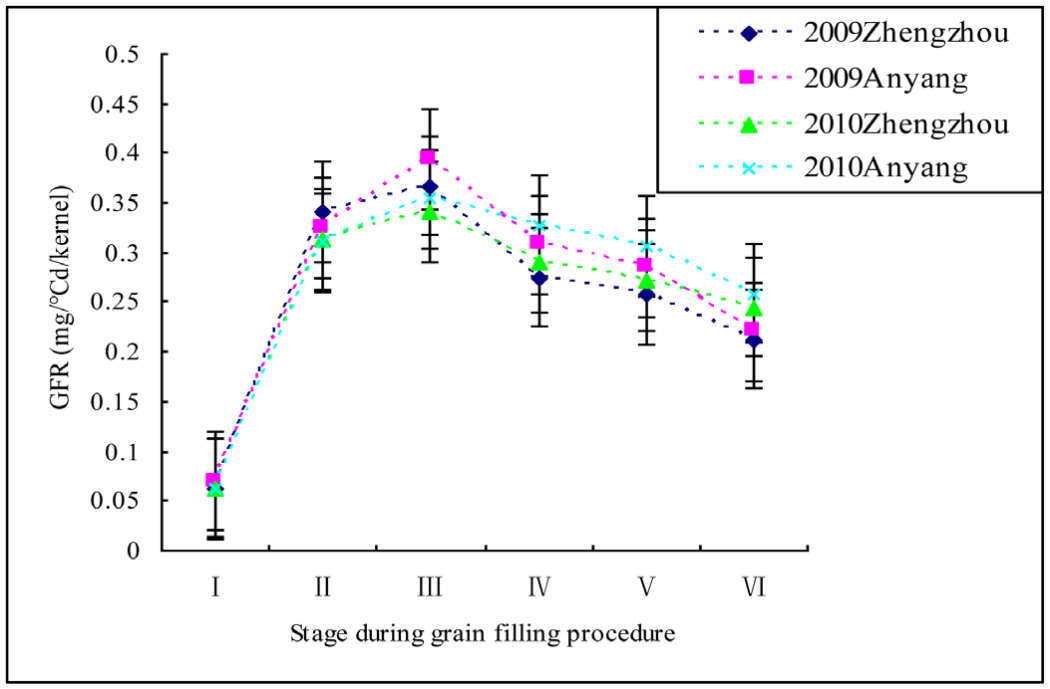
Dynamic diversity of grain filling rates in maize strains.

Among the IF_2_ population (Table 1; Fig. 2), the GFR at the six sampling stages at Anyang were higher than the corresponding sampling stage at Zhengzhou in 2009 and 2010, respectively. However, the GFR during the grain filling process over a year showed a similar tendency at both locations, and the variations in the GFR among the population increased mainly in the middle or later stages (23–50 DAP). Under different environments, there were no significant variations in the KW of the IF_2_ population; however, the GFD between the 2 years was significantly different at both locations. The dynamic diversification of GFR in all materials shows a tendency for logistic curves: the GFR gradually rose in the first and second sampling stages, reaching a maximum at the third sampling stage for different years or locations.

In the table 2, the GFR and KW were significantly positively correlated, except at the first sampling stage, which confirmed that the variance of KW is associated with the GFR during the effective grain filling period. Moreover, there were extremely significant positive correlations between sampling stage II (16–22 DAP) and KW, indicating that 16–22 DAP is an important stage for determining the final kernel weight. There was no significant correlation between GFR and GFD.

**Table 2:** Correlation coefficients between grain filling rate, grain filling duration and final kernel weight.

Location		I	II	III	IV	V	VI	KW	GFD
Zhengzhou	I		−0.1	−0.07	−0.17	0.15	0.25	0.23	0.11
	II	0.02		−0.29*	0.08	0.01	0.03	0.40**	0.23
	III	−0.21	−0.32*		−0.05	0.04	0.09	0.30*	−0.10
	IV	0.17	−0.02	−0.22		−0.23	−0.05	0.31*	−0.15
	V	−0.21	0.03	0.03	−0.23		0.02	0.32*	−0.22
	VI	−0.01	0.03	0.36*	0.01	−0.08		0.31*	0.05
	KW	−0.01	0.42**	0.48**	0.31*	0.1	0.39**		0.08
	GFD	0.07	0.17	−0.03	0.05	−0.09	0.04	0.15	
Anyang	I		−0.24	0.01	0.09	−0.07	0.24	0.31*	0.04
	II	−0.26		−0.37*	0.05	0.2	0.06	0.39**	−0.02
	III	−0.07	−0.35*		−0.04	−0.21	−0.17	0.02	0.09
	IV	−0.16	0.21	−0.15		−0.18	−0.13	0.52**	−0.12
	V	−0.13	0.08	0.14	−0.12		−0.21	0.36*	−0.06
	VI	0.02	0.2	0.02	0.01	−0.12		0.33*	−0.27
	KW	0.04	0.52**	0.55**	0.31*	0.36*	0.51**		−0.24
	GFD	0.11	−0.04	0.11	0.05	−0.17	−0.15	0.06	

Note: *, **Significant effect at probabilities of 0.05 and 0.01, respectively;

The correlation coefficients for 2009 are in the upper triangular area of the table and the correlation coefficients for 2010 are in the lower triangular area of the table.

### Unconditional QTLs Detected for Grain Filling Rate

The genetic linkage map for the recombinant inbreed line (RIL) population was constructed using 217 SSR markers, which included 10 linkages, and spanned 2438.2 cM, with an average interval of 11.2 cM. [26] The genotypes of each cross of the IF_2_ population were deduced from the marker genotypes of their RIL parents, and the molecular linkage map for QTL mapping in the IF_2_ population was used as the molecular linkage map of the RIL population because it had the same genetic background. [27].

**Figure 3:**
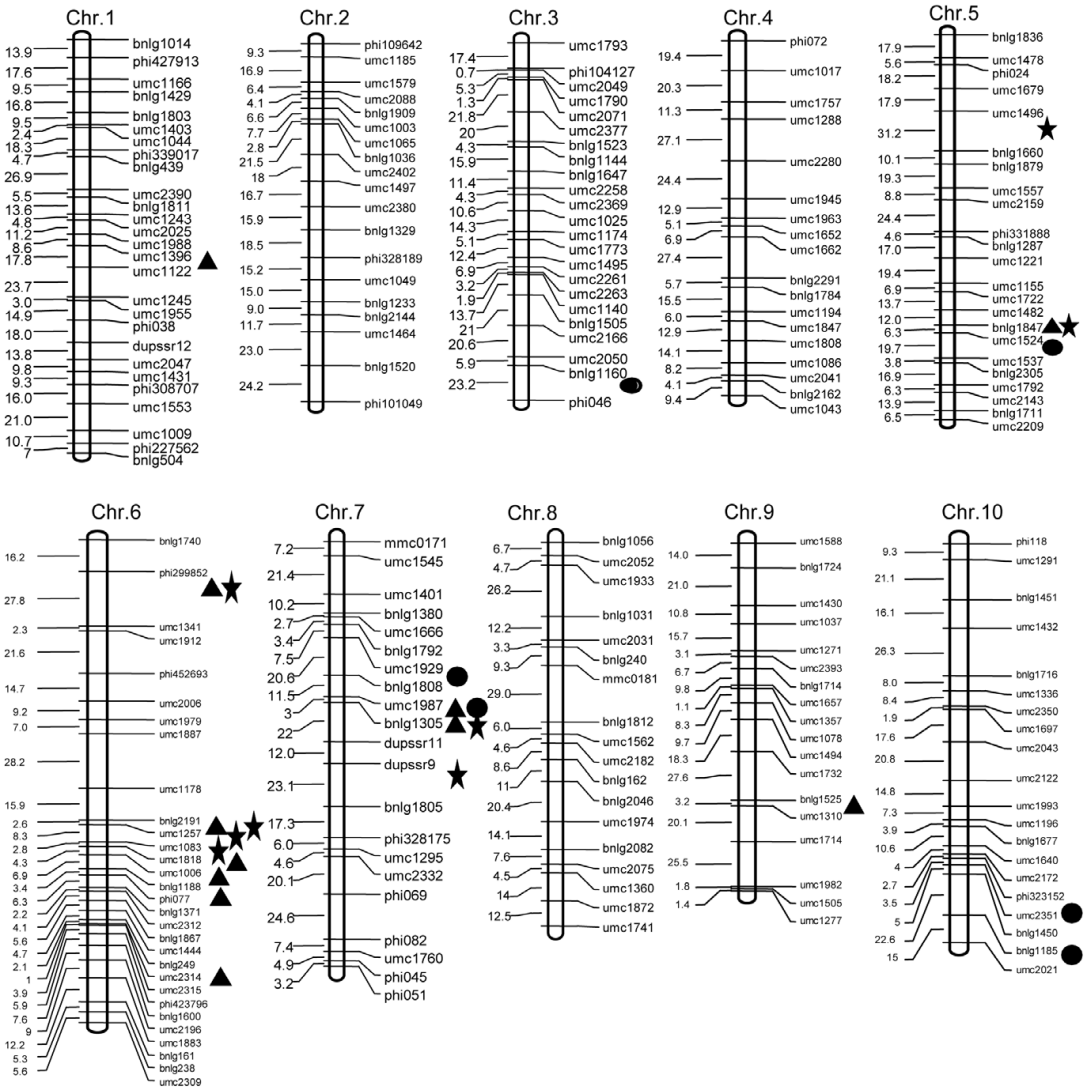
Chromosomal locations of QTLs detected for grain filling rate and kernel weight. Note: *Triangle* unconditional QTLs detected for grain filling rate, *Star* conditional QTLs detected for grain filling rate, *Round* unconditional QTLs detected for kernel weight.

Thirty-three different unconditional QTLs were identified for the GFR at the six sampling stages between 2 years at two locations, and were located on five chromosomes (Table 3; Fig. 3). In the four environments, there were 11, 9, 7 and 7 QTLs detected, respectively. Among them QTLs *qGFR7b*, *qGFR9* and *qGFR6d* were identified at the same stages at both locations over 2 years. Moreover, QTL *qGFR7b* was detected at sampling stage II (16–22 DAP) and stage VI (44–50 DAP). QTL *qGFR9*, derived from the parent Xu 178, showed 7.51%, 6.67%, 6.67% and 7.01% of total variance at the two locations over two years, respectively. Another QTL, *qGFR6d,* identified at the fifth stage (37–43 DAP), could explain 5.31%, 5.32%, 5.49% and 5.42% of the total phenotypic variance, respectively. However, QTL *qGFR6d,* coming from the parent Huang-C and detected at stage IV (29–36DAP), could explain 21.14% of the total variance. QTLs *qGFR7a*, *qGFR6a* and *qGFR6c* were detected at the same stages in the four environments, except at Anyang in 2010. QTL *qGFR7a*, identified at stage II (16–22 DAP), contributed 10.96%, 11.92% and 13.27% of the total variance, respectively. QTL *qGFR6a* was detected at stage I (0–15DAP) and could explain 33.85%, 23.3% and 19.83% of the total phenotypic variance, with a direct increase of 0.021, 0.016 and 0.013 mg °Cd^−1^ kernel^−1^ of GFR, respectively. QTL *qGFR6c*, detected at stage IV (37–43DAP), contributed 24.37%, 20.75% and 21.67% of the total phenotypic variance, with a direct increase of 0.067, 0.064 and 0.063 mg °C d^−1^ kernel^−1^ of GFR, respectively.

**Table 3:** Unconditional QTLs detected for grain filling rate in the immortalized F_2_ population.

Year	Location	Stage/Trait	QTL^a^	Markers interval	LOD^b^	A^c^	D^c^	Effects^d^	R^2e^(%)
2009	Zhengzhou	I	*qGFR5*	bnlg1847-umc1524	4.67	0.012	−0.006	PD	22.05
		I	*qGFR6a*	phi299852-umc1341	4.44	0.014	−0.005	PD	33.85
		II	*qGFR7a*	umc1987-bnlg1305	5.12	0.043	0.005	A	10.96
		II	*qGFR7b*	bnlg1305-dupssr11	3.72	0.041	−0.003	A	10.96
		III	*qGFR9*	bnlg1525-umc1310	4.29	−0.029	0.058	OD	7.51
		IV	*qGFR6b*	umc1006-bnlg1188	4.49	0.084	−0.042	PD	25.04
		IV	*qGFR6c*	phi077-bnlg1371	4.25	0.085	−0.051	PD	24.37
		IV	*qGFR6d*	umc1818-umc1006	4.16	0.077	−0.030	PD	21.14
		IV	*qGFR6e*	umc2315-phi423796	4.07	0.063	−0.055	D	13.52
		V	*qGFR6d*	umc1818-umc1006	5.27	−0.035	0.077	OD	5.31
		VI	*qGFR7b*	bnlg1305-dupssr11	4.44	0.086	−0.028	PD	22.91
		KW	*qKW7a*	umc1987-bnlg1305	7.46	0.020	0.003	A	10.59
		KW	*qKW10a*	bnlg1185-umc2021	4.72	0.015	−0.007	PD	14.94
		KW	*qKW3a*	bnlg1160-phi046	3.96	−0.015	−0.004	PD	12.07
		KW	*qKW7b*	umc1929-bnlg1808	3.92	−0.025	0.006	PD	17.99
	Anyang	I	*qGFR6a*	phi299852-umc1341	4.07	0.012	−0.006	PD	23.3
		II	*qGFR7a*	umc1987-bnlg1305	4.29	0.051	−0.002	A	11.92
		II	*qGFR7b*	bnlg1305-dupssr11	3.92	0.056	−0.016	PD	15.73
		III	*qGFR9*	bnlg1525-umc1310	4.09	−0.027	0.056	OD	6.67
		IV	*qGFR6b*	umc1006-bnlg1188	4.39	0.088	−0.045	PD	25.17
		IV	*qGFR6e*	umc2315-phi423796	4.08	0.063	−0.059	D	12.61
		IV	*qGFR6c*	phi077-bnlg1371	3.83	0.082	−0.054	PD	20.75
		V	*qGFR6d*	umc1818-umc1006	5.09	−0.035	0.076	OD	5.32
		VI	*qGFR7b*	bnlg1305-dupssr11	3.94	0.061	−0.008	A	15.42
		KW	*qKW10a*	bnlg1185-umc2021	5.49	0.016	−0.004	PD	16.64
		KW	*qKW5*	umc1524-umc1537	4.91	−0.017	−0.001	A	17.34
2010	Zhengzhou	I	*qGFR6a*	phi299852-umc1341	3.78	0.010	−0.009	D	19.83
		II	*qGFR7a*	umc1987-bnlg1305	4.11	0.041	−0.004	A	13.27
		II	*qGFR7b*	bnlg1305-dupssr11	4.07	0.052	−0.028	PD	21.73
		III	*qGFR9*	bnlg1525-umc1310	4.07	−0.027	0.056	OD	6.67
		IV	*qGFR6c*	phi077-bnlg1371	3.8	0.081	−0.050	PD	21.67
		V	*qGFR6d*	umc1818-umc1006	4.54	−0.036	0.074	OD	5.49
		VI	*qGFR7b*	bnlg1305-dupssr11	4.7	0.085	−0.021	PD	22.08
		KW	*qKW7a*	umc1987-bnlg1305	6.68	0.021	0.002	A	12.65
		KW	*qKW7b*	umc1929-bnlg1808	4.98	−0.021	0.003	A	18.92
		KW	*qKW10b*	phi323152-umc2351	4.15	0.013	−0.005	PD	10.71
	Anyang	I	*qGFR1*	umc1396-umc1122	3.98	−0.003	0.013	OD	15.03
		II	*qGFR5*	bnlg1847-umc1524	5.09	−0.048	−0.019	PD	16.39
		II	*qGFR7b*	bnlg1305-dupssr11	4.29	0.052	−0.028	PD	22.03
		III	*qGFR9*	bnlg1525-umc1310	4.28	−0.028	0.057	OD	7.01
		IV	*qGFR6f*	bnlg2191-umc1257	4.19	0.024	−0.060	OD	5.88
		V	*qGFR6d*	umc1818-umc1006	4.6	−0.032	0.075	OD	5.42
		VI	*qGFR7b*	bnlg1305-dupssr11	4.85	0.088	−0.022	PD	23.3
		KW	*qKW7a*	umc1987-bnlg1305	6.54	0.018	0.002	A	10.45
		KW	*qKW10a*	bnlg1185-umc2021	4.24	0.014	−0.007	PD	13.53

Note: ^a^Unconditional QTLs detected for grain filling rate in the IF_2_ population;

b Logarithm of Odds for each QTL.

c A, additive values; D, dominant values;

d The effect of each QTL; A, additive; PD, partial dominance; D, dominance; OD, overdominance;

e R^2^ contribution rate.

There were eleven QTLs detected for kernel weight, and were located on four chromosomes at the two locations over 2 years (Table 3; Fig. 3). The QTL *qKW10a* was detected at both locations in 2009 and at Anyang location in 2010, and contributed 14.94%, 16.64%, and 13.53% of total phenotypic variance, respectively. The QTL *qKW7a* was detected at Zhengzhou in 2009 and at both locations in 2010 and contributed 10.59%, 12.65% and 10.45% of total variance, respectively. In addition, the *qGFR7a* was co-located with the QTL *qKW7a* at Zhengzhou location over 2 years.

### Conditional QTL Mapping for Grain Filling Rate

Fourteen conditional QTLs were detected at five stages for the GFR and are distributed on chromosomes 6 and chromosome 7 (Fig. 3; Table 4). These QTLs clustered at chromosome bins 6.01–6.02 and 7.02–7.03, which correlates with the unconditional QTL mapping results. The conditional QTL *qGFR7c*, identified at stage V|IV (37–43DAP) at two locations over 2 years, contributed 14.77%, 18.70%, 21.40% and 20.08% of the total phenotypic variance in GFR. QTL *qGFR7b*, detected at the sixth stage (44–50DAP) at Zhengzhou in both years and Anyang in 2010, could explain 14.55%, 10.04% and 10.17% of the total variance, respectively. In addition, QTL *qGFR7b*, identified at stage II|I (0–15DAP) at Anyang in 2010, could explain 17.81% of the total variance in the GFR. In 2009, QTL *qGFR6g* was detected at two locations, contributing 11.82% and 14.42% of the total variance, respectively. In 2010, QTL *qGFR6h,* derived from the parent Huang-C, was detected at two locations, and contribution large proportions of the phenotypic variance: 38.42% and 37.27%, respectively.

**Table 4:** Conditional QTLs detected for grain filling rate in the immortalized F_2_ population.

Year	Location	Stage	QTL^a^	Markers interval	LOD^b^	A^c^	D^c^	Effects^d^	R^2e^(%)
2009	Zhengzhou	I	*qGFR5a*	bnlg1847-umc1524	4.65	0.012	−0.006	PD	21.88
		I	*qGFR6a*	phi299852-umc1341	4.49	0.023	−0.005	PD	33.85
		IV|III	*qGFR6g*	umc1257-umc1083	3.67	0.048	−0.066	OD	11.82
		V|IV	*qGFR7c*	dupssr9-bnlg1805	3.87	0.059	−0.065	D	14.77
		VI|V	*qGFR7b*	bnlg1305-dupssr11	6.07	0.058	0.023	PD	14.55
	Anyang	I	*qGFR6a*	phi299852-umc1341	4.03	0.012	−0.006	PD	23.18
		IV|III	*qGFR6g*	umc1257-umc1083	3.66	0.055	−0.066	OD	14.42
		V|IV	*qGFR7c*	dupssr9-bnlg1805	4.97	0.066	−0.072	D	18.70
2010	Zhengzhou	I	*qGFR6a*	phi299852-umc1341	3.72	0.010	−0.009	D	19.25
		IV|III	*qGFR6f*	bnlg2191-umc1257	5.56	0.025	−0.076	OD	6.42
		IV|III	*qGFR6h*	umc1083-umc1818	4.07	0.117	−0.054	PD	38.42
		V|IV	*qGFR7c*	dupssr9-bnlg1805	5.87	0.071	−0.073	D	21.40
		VI|V	*qGFR7b*	bnlg1305-dupssr11	4.59	0.049	0.032	PD	10.04
	Anyang	I	*qGFR1*	umc1396-umc1122	3.95	−0.003	0.013	OD	15.03
		II|I	*qGFR7b*	bnlg1305-dupssr11	4.38	0.062	−0.014	PD	17.81
		IV|III	*qGFR6f*	bnlg2191-umc1257	4.71	0.022	−0.075	OD	5.33
		IV|III	*qGFR6h*	umc1083-umc1818	4.11	0.117	−0.056	PD	37.27
		V|IV	*qGFR7c*	dupssr9-bnlg1805	5.11	0.069	−0.070	D	20.08
		VI|V	*qGFR7b*	bnlg1305-dupssr11	4.84	0.049	0.033	PD	10.17

Note: ^a^Conditional QTLs detected for grain filling rate in the IF_2_ population;

b Logarithm of Odds for each QTL.

c A, additive values; D, dominant values;

d The effect of each QTL; A, additive; PD, partial dominance; D, dominance; OD, overdominance;

e R^2^ contribution rate.

Comparing the results of the unconditional and conditional QTL mapping methods (Table 3; Table 4; Fig. 3), there were five unconditional QTLs detected under conditional mapping in the same environments. At the sixth stage (44–50 DAP), QTL *qGFR7b* was identified by both QTL mapping methods in all four environments, except at Anyang in 2009 under conditional QTL mapping. *qGFR7b* showed higher effects (22.91%, 22.08% and 23.30% of the total variance) under unconditional QTL mapping, than under conditional QTL mapping (14.55%, 10.04% and 10.17% of the total variance). At the second stage (16–22 DAP), *qGFR7b* was identified at Anyang in 2010 using both QTL mapping methods, and contributed 22.03% and 17.81% of the total variance. Additionally, QTL *qGFR6f* was identified at the fourth sampling stage (30–36 DAP) under both methodologies at Anyang in 2010. Among the new QTLs detected by conditional QTL mapping, *qGFR6g* and *qGFR6h* were adjacent to the unconditional *qGFR6b* and *qGFR6d* on chromosome 6, and *qGFR7c* was located at the adjacent locus to the unconditional QTLs *qGFR7a* and *qGFR7b* on chromosome 7.

## Discussion

In maize, many previous studies on grain filling or kernel development used several inbred lines and hybrids (with different genetic backgrounds), or RIL populations for QTL mapping. [4], [16], [26], [28] The GFR is easily affected by meteorological factors, edaphic conditions, water and fertilizer management levels, as well by plant density. [29]–[32] Comparing with inbred lines and RIL populations, [4], [26] an IF_2_ population not only has similar heterotic phenotypes to hybrid maize, which are not easily affected by various environmental factors, but also each family of the IF_2_ population has similar flowering and silking times. Thus, using an IF_2_ population ensured accurate phenotypic values for the GFR in this study.

As in previous reports, the GFR and GFD were determined by genotype and were influenced by environmental factors. [29]–[32] Stewart et al. reported that when maize is grown under a very broad range of temperatures, plant development in response to temperature is nonlinear during the reproductive period. [29] When grown over a narrower range of temperatures, the response reported by Stewart et al. approximated a linear relationship, with the base temperature near 0°C. [29], [30] In addition, because of the narrower range of temperature encountered in this study, the GFR was evaluated using heat units between sampling times, and daily °C d values for grain filling were measured at the base temperature of 0°C. Using this method, Borrás and Otegui evaluated the effective grain filling rate using two hybrids, [33] and the kernel growth rate was also measured for two hybrids and a set of inbred lines by Borrás et al. [12], [15], [28] In the previous study of Liu et al., a set of RIL population was adopted for identifying GFR related QTL in maize, days between two sampling times were used as grain filling duration for calculating GFR. [26] In this study, thermal time between two sampling times were used for evaluating GFR value, which could benefit of decreasing the affects of temperature.

Kernel development is a complex process with a dynamic character that is regulated by three physiological activities during the reproductive period: (1) cell division and differentiation; (2) the effective grain filling period, and (3) the maturation drying period. [34] The GFR is low speed during the cell division and differentiation phase, during which almost no dry matter accumulates. [19] The effective grain filling period is a process of rapid dry matter accumulation resulting from the deposition of seed reserves. In this period, the GFR rises gradually and reaches its maximum value in the middle of the period. [15] During the maturation drying phase, the GFR decreases gradually, with kernels continuing to lose water. Here, six samplings during effective grain filling period and the maturation drying phase (15–50 DAP) were adapted for GFR evaluation that is because of the dry matter mainly accumulate in the two periods. And, starch synthesis in the kernel begins from 12–15 DAP. [35] In this study, the GFR of the IF_2_ population gradually rose in the first and second stages, reaching a maximum at the third stage, and then gradually decreased over the last three stages (Fig. 2).

Grain filling determines the final kernel weight, and thus contributes greatly to grain productivity. It is reported that the variation in KW may be achieved through different combinations of kernel growth rates and grain filling durations; however, there was no correlation between kernel growth rate and grain filling duration. [16], [36] In the present study, there was no significant correlation between the correlation between the GFR and GFD. However, in this study, the variation in KW was determined by the GFR during the effective grain filling period and maturation drying stage, and there was no correlation between KW and the GFD.

Although physiologists have directed their attention to the grain filling processes, there have been few genetic studies of grain filling because of its complex and dynamic features. [10] Wang et al. performed a genetic analysis on the GFR and GFD in maize, and their results revealed that general combining ability (GCA) was more important than special combining ability (SCA) for both the GFR and the effective filling duration. [13] QTL mapping for grain filling using a RIL population in maize was reported by Thévenot et al. for enzyme activities and soluble carbohydrates, [4] and by Liu et al. for the GFR. [26] Thévenot et al. reported that a higher density QTLs was detected on chromosome 1 and 2 at 35 DAP, and that QTLs were detected that clustered at bin 5.02–5.03 and 5.04–5.05 at 15 DAP. [4] In this study, a higher density of QTLs was identified on chromosome 6 at 30–36 DAP, clustered at bin 6.01–6.02. However, there is still a number of QTLs for GFR that co-localize to the QTLs at same chromosomal bin for enzyme activities, soluble carbohydrates, and the genes associated with grain filling. [4] For example, *qGFR1* is located at the same chromosomal bin as the *BT2* gene, a QTL for fresh matter and a QTL for neutral-cytosolic invertase. QTL *qGFR5* was identified at stage 0–15 DAP in the same chromosomal fragment as a QTL for glucose, fructose and sucrose content; and QTL *qGFR6a* was identified in the same chromosomal fragment as the *BT1* gene and a QTL for glucose content. In addition, QTL *qGFR9* was located in the same chromosomal bin as a QTL for sucrose synthase, glucose content and fructose content. QTLs *qGFR7a, qGF7b* and *qGFR7c* co-localize with a QTL for glucose at chromosomal bin 7.02–7.03. At chromosomal bin 6.01–6.02, there were the gene *6PGDH* (6-phosphogluconate dehydrogenase) and a QTL for fresh matter co-localize. These results reveal that the grain filling process not only involves starch synthesis, but also other novel activities.

Grain filling represents a process of starch accumulation, [2] and there have been many reports of the starch pathway in cereals. For example, in rice Ohdan et al. analyzed the genes associated with starch synthesis at the level of transcription during the grain filling process. [37] They divided the 27 starch synthesis-associated genes into four groups. Group 1 genes are expressed very early in grain formation and are presumed to be involved in the construction of fundamental cell structure and de novo synthesis of glucan primers. Group 2 genes are highly expressed throughout the grain development process. Group 3 genes are transcribed at a low level at the onset, but rise steeply at the beginning of starch synthesis in the endosperm. Group 4 genes are barely expressed, mainly at the onset of grain development. Group 3 genes are thought to play essential roles in endosperm starch synthesis. Yan et al. compared the starch synthesis genes between maize and rice, and detected thirty starch synthesis genes in the maize genome, which covered all the starch synthesis gene families encoded by 27 genes in rice. [38] Among the unconditional QTLs detected for the GFR in this study, QTL *qGFR6a* was only identified at the first stage in three out of four environments; this kind of QTL resembles a group 1 and group 4 gene of starch synthesis. [37] However, no QTL was detected for the GFR that was expressed throughout the whole process of grain filling. These results indicated that the GFR is regulated by genes that are selectively expressed at different grain filling stages. Among these QTLs identified for GFR, QTL *qGFR7b* was identified at different stages in four environments using two QTL mapping methods; therefore, it represents a main QTL for the GFR. In addition, several QTLs, such as *qGFR6a*, *qGFR6d*, *qGFR9*, *qGFR6c*, *qGFR6f* and *qGFR7c*, were detected in different environments, and might represent genes with important effects in regulating grain development. Several QTLs were identified in single environments and stages, which might be caused by the differences in climate factors under the different environments and grain filling stages. Although, thermal time was the main contributor to GFR, the other climate factors also had a certain influence to grain filling rate and grain filling duration. [12], [15], [31], [32] In this study, the average temperature and daily sunlight were significant different (Fig. 1a and 1b) between the two locations. And, there were large differences at according stages in related climate factors between the two years at any one location. So in this study, the thermal time was used as for calculating GFR, and used as input data for QTL mapping However, under the affects of the other different environmental factors, most unconditional and conditional QTLs for GFR expressed selectively.

In recent decades, increases in grain yield in maize were achieved mainly by lengthening the grain filling period and increasing population density, which in turn increased GFR per unit land area. GFD was longer in the newer hybrids; even though harvest maturity remained unchanged. [36] The increase in GFD was the result of delayed physiological maturity rather than a change in flowering date. The GFR is somewhat more stable than GFD, and the latter is easily affected by changes in plant density and temperature, whereas the kernel growth rate is not affected. [13] Additionally, the KW is associated with the GFR during the effective grain filling period, as reported in this study. In many countries or areas of the world, the season for maize growth is very limited, and the tendency for use of mechanical harvesting demands hybrid maize with a relatively short period of dehydration in the field. Thus, commercial hybrids must have a high GFR and an appropriate growth duration to obtain high grain yields.

## Materials and Methods

### The Development of the Immortalized F_2_ Population

A population of 166 RILs was constructed by a single-seed descent method from two elite inbred lines, Huang-C and Xu178. The cross was an elite hybrid, Nongda108, which occupied approximately 2.7 million hectares during 2001–2004 in China. One of its parents, Huang-C, was selected from Chinese germplasm, and the other parent, Xu178, was derived from an exotic hybrid. According to the procedure described by Hua et al., [39] the 166 RILs were randomly divided into two groups, each group including 83 RILs. Then, pairs of crosses were made randomly between the lines of the two groups, without repetition, so that 83 different crosses were generated. The procedure was repeated three times. Finally, 249 (83×3) pairs of crosses between the two RILs formed the immortalized F_2_ population. Six crosses lacked abundant seeds because of a difficulty in mating; thus, 243 crosses were used in this study.

### Field Evaluation

The IF_2_ population, the two parents, and the hybrid were planted in 2009 and 2010 on the Agronomy Farm of Henan Agricultural University (Zhengzhou, 113°42′E, 34°48′N), which is located in the central region of China and has an average daily temperature 14.3°C and an average annual rainfall of 640.9 mm. The maize plants were also planted during the same years at the Anyang Agricultural Institute (Anyang, 114°21′E, 36°6′N), which is located in the center of the north China plain and has an average temperature of 14.1°C and an average of 556.9 mm of rainfall per year. At Zhengzhou, all the plant materials were planted on the 12th and 8th of June in 2009 and 2010, respectively. At Anyang, plant materials were planted on 17th and 12th of June in 2009 and 2010, respectively. The field experimental design followed an incomplete block design approach, with two replications at each location. Each experimental material was applied to two plots of 6 m long×0.67 m wide rows and comprised 50 plants, at a density of 65,250 plants per hectare. The fields were kept free of weeds and nests, and irrigated and fertilized properly to avoid nutritional stress.

### Sampling and Measurements of GFR

In each plot, when 50% of the silks spit out of all plants, the pollination date was determined. Samples were hand-collected for five ears at each plot at 15, 22, 29, 36, 43 and 50 days after pollination (DAP) in 2009 and 2010, respectively. The sampling dates were chosen starting at 15 DAP because previous studies have shown that starch synthesis in the kernel begins from 12–15 DAP. [35] Ears with irregular kernel sets along the ear row were discarded to avoid the confounding effect of atypically large kernels adjacent to unpollinated florets. [12] These harvested ears were dried fully under nature condition, and the grains in the center of the ear were threshed. The moisture content of all the grain samples was detected by PM-8188NEW grain moisture determination apparatus. And, the grain moisture values for all the samples were amended to 13%, and then the 100-kernel weight was evaluated. These treatments were used for ensuring all the samples harvested at different grain filling stages in the same moisture. The 100-kernel weight in the center of the ear was then quantified three times, and the average data among the three 100-kernel weights for every sampling time were calculated. The GFR between two sampling stages was calculated as: GFR (mg °Cd^−1^ kernel^−1^) = the margin of kernel weight for two sampling times (mg kernel^−1^)/GFD between two sampling times (°Cd). The GFR of pollination date-15DAP (I), 16–22 DAP (II), 23–29DAP (III), 30–36DAP (IV), 37–43DAP (V) and 44–50DAP (VI) were calculated, respectively. Here, we used thermal time as the GFD, [12], [14], [28] which is calculated using the daily air temperature values between two sampling times. In addition, the daily °Cd value for grain filling was calculated using 0°C as base temperature. [29] The average performance data generated in each replication and location were used as raw data for further analyses. Data analysis was performed using SAS 9.2 statistical software package with the PROC MIXED procedure. [40] The climate data were obtained from the Climate Bureau of Zhengzhou and the Climate Bureau of Anyang, China.

### Unconditional and Conditional QTL Mapping

Unconditional QTL mapping was performed using the composite interval mapping method and Model 6 of the Zmapqtl module of QTL Cartographer 2.5. [41] The threshold of a logarithm of Odds (LOD) was calculated using 1,000 permutations at a significance level of P = 0.05, with scanning intervals of 2 cM between markers and a putative QTL, and a 10 cM window. The number of marker cofactors for background control was set by forward-backward stepwise regression with five controlling markers.

For dynamic traits of developmental behavior, the genetic effect (G_(*t*)_) at time *t* is the genetic effect (G_(*t−1*)_) at time (*t−1*) and the extra genetic effect (G_(*d*)_). [42]–[44] Thus, it calculates the cumulative gene effects from initial time to *t*, but not for the independent effects of gene expression in the duration (*t−1*) to *t*. To reject the genetic effect of a genetic effect (G_(*t−1*)_) at time *t*, the conditional phenotypic values y _(t |t−1)_ were obtained by the mixed model approach for the conditional analysis of quantitative traits described by Zhu. [42] The conditional phenotypic values were used as input data for conditional QTL mapping, which used the composite interval mapping method.
